# Thyroid hormone receptor binding to DNA and T_3_-dependent transcriptional activation are inhibited by uremic toxins

**DOI:** 10.1186/1478-1336-3-1

**Published:** 2005-04-04

**Authors:** Guilherme M Santos, Carlos J Pantoja, Aluízio Costa e Silva, Maria C Rodrigues, Ralff C Ribeiro, Luiz A Simeoni, Noureddine Lomri, Francisco AR Neves

**Affiliations:** 1 Molecular Pharmacology Laboratory, Department of Pharmaceutical Sciences, School of Health Sciences, University of Brasilia, Brazil; 2 SOCLIMED – Dialysis Unit, Brasília, Brazil; 3 University of Cergy-Pontoise, UFR des Sciences et Techniques, ERRMECe Laboratory, BP222, 2 Ave Adolphe Chauvin, 95302 Cergy-Pontoise, France

## Abstract

**Background:**

There is a substantial clinical overlap between chronic renal failure (CRF) and hypothyroidism, suggesting the presence of hypothyroidism in uremic patients. Although CRF patients have low T_3 _and T_4 _levels with normal thyroid-stimulating hormone (TSH), they show a higher prevalence of goiter and evidence for blunted tissue responsiveness to T_3 _action. However, there are no studies examining whether thyroid hormone receptors (TRs) play a role in thyroid hormone dysfunction in CRF patients. To evaluate the effects of an uremic environment on TR function, we investigated the effect of uremic plasma on TRβ1 binding to DNA as heterodimers with the retinoid X receptor alpha (RXRα) and on T_3_-dependent transcriptional activity.

**Results:**

We demonstrated that uremic plasma collected prior to hemodialysis (Pre-HD) significantly reduced TRβ1-RXRα binding to DNA. Such inhibition was also observed with a vitamin D receptor (VDR) but not with a peroxisome proliferator-activated receptor gamma (PPARγ). A cell-based assay confirmed this effect where uremic pre-HD ultrafiltrate inhibited the transcriptional activation induced by T_3 _in U937 cells. In both cases, the inhibitory effects were reversed when the uremic plasma and the uremic ultrafiltrate were collected and used after hemodialysis (Post-HD).

**Conclusion:**

These results suggest that dialyzable toxins in uremic plasma selectively block the binding of TRβ1-RXRα to DNA and impair T_3 _transcriptional activity. These findings may explain some features of hypothyroidism and thyroid hormone resistance observed in CRF patients.

## Background

Chronic renal failure (CRF) is associated with disturbances in the internal milieu, with repercussions on the immune, hematopoietic, gastrointestinal and endocrine systems and organs [1-4]. Patients with advanced CRF display a variety of hormonal abnormalities including perturbations of the hypothalamic-pituitary-thyroid hormone endocrine axis. The peripheral thyroid hormone metabolism is also altered in patients with CRF [5-9].

In uremia various thyroid hormone physiological characteristics are altered. Total and free tyroxine (T_4_) and 3,5,3'-triiodothyronine (T_3_) levels in the serum are frequently reduced in patients with CRF [10,11]. Reduced T_3 _levels might be explained by decreased peripheral tissue conversion of T_4 _to T_3 _[12]. Most CRF patients, however, are considered to be euthyroid as evidenced by normal thyroid-stimulating hormone (TSH) levels [11,13]. In addition, the prevalence of thyroid diseases, including goiter and hypothyroidism, are also higher in CRF patients than in the general population [10].

Thyroid hormones control numerous aspects of mammalian development and metabolism, of which most of these actions are mediated by specific thyroid hormone receptors (TRs). An important metabolic activity of thyroid hormones is to increase oxygen consumption of target tissues [14,15]. In fact, in experimental renal failure and in uremic patients the expected increase in basal oxygen consumption following the administration of T_3 _is not observed, suggesting that CRF is associated with resistance to thyroid hormone action [16-18]. However, it is currently not known whether thyroid hormone receptors play a role in the thyroid dysfunction observed in uremic patients.

TRs are ligand-regulated transcription factors of the nuclear receptor superfamily which includes steroid hormones and vitamin D receptors and also PPARγ [15,19,20]. TRs modulate gene expression by binding specific DNA sequences, known as thyroid response elements (TREs), found in the promoters of TR-regulated genes. TREs are composed of repeats of the consensus half-site AGGTCA in a variety of different orientations, including direct repeats spaced by four nucleotides (DR-4), inverted palindromes (F2) and palindromes [21,22]. In the presence of T_3_, TRs preferentially form heterodimers with the retinoid X receptors (RXRs) although unliganded TR binds to DNA as either homodimers or monomers [20,23].

In recent years, it has become apparent that uremic toxins can impair the function of some nuclear receptors, such as the vitamin D receptor (VDR). Previous studies suggest that uremic toxins inhibit the binding of VDR to DNA and can contribute to the vitamin D resistance observed in CRF patients [24-28]. It is, therefore, conceivable that uremia also induces modifications in thyroid hormone receptors and consequently plays a role in the thyroid hormone dysfunction observed in uremic patients.

We investigated the effects of uremia on TRβ1 function by studying the ability of TRs to bind to DNA sequences in the presence or absence of uremic plasma collected from CRF patients. Our results showed that uremic plasma significantly reduced the binding of TR heterodimers (TRβ1-RXRα), but not of homodimers (TRβ1-TRβ1) to DR-4. Furthermore, uremic plasma also inhibited the binding of a VDR heterodimer (VDR-RXR) to DR-3, while the binding of PPARγ to DR-1 remained unaltered. Moreover, hemodialysis (HD) diminished the inhibitory effect of CRF patients' plasma on the binding of both TR and VDR heterodimers to DNA. When human promonocyte cells were incubated with ultrafiltrate collected Pre-HD the transcriptional activation induced by T_3 _was inhibited. This inhibition was lost when the cells were treated with ultrafiltrate collected after-HD. Thus, we suggest that dialyzable uremic toxins selectively block the binding of TRβ1-RXRα and VDR-RXR heterodimers to DNA and reduce the transcriptional activities regulated by these receptors. These results indicate that the thyroid hormone dysfunction observed in uremia may be partially explained by T_3 _resistance induced by impaired TRβ1 function.

## Results

### Uremic plasma inhibits the binding of hTRβ1-hRXRα on DR-4

**Figure 1:**
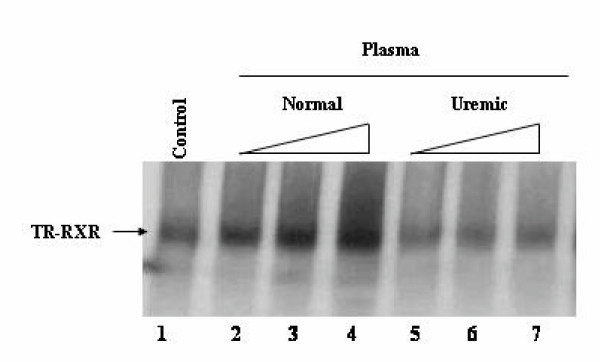
**Uremic plasma reduces hTRβ1-hRXRα complex formation on DR-4. **Gel Shift experiments were performed using in vitro translated [^35^S] hTRβ1, cold hRXRα and DR-4. [^35^S] hTRβ1 was treated with T_3 _10^-7^M for 30 min at 4°C and then incubated without (Control – lane 1) or with increasing volumes (0.5, 1.0, 2.0 μL) of normal (lanes 2–4) or uremic (lanes 5–7) plasma for 30 min at 4°C. Cold DR-4 type TRE (5'- AGCT TC **AGGTCA **CAGG **AGGTCA **GAG - 3'), cold hRXRα, and nonspecific DNA poly (dIdC) were subsequently added and incubated for 20 min.

To study the effects of uremic plasma on the ability of TRβ1 to bind to a specific TRE we analyzed the binding of hRXRα-hTRβ1 heterodimers to DR-4. In this assay, the protein-DNA complex was visualized by labeling the TRβ1 with ^35^S. In the presence of T_3_, the addition of increasing amounts (Figure 1; lanes 2–4) of plasma from normal individuals improved the binding of hRXRα-hTRβ1 to DNA. Conversely, TR incubation with uremic plasma (lanes 5–7) collected prior to hemodialysis significantly reduced the binding of heterodimers (RXR-TR) to DR-4. Band densitometry analysis of 5 independent experiments showed that uremic plasma reduced hRXRα-hTRβ1-DR-4 complex formation by 77 ± 15%, compared to plasma from normal subject (not shown). Similar results were observed for the thyroid response element F2 (inverted palindrome) in which uremic plasma also inhibited the RXR-TR binding to DNA (not shown). Pre-treatment of hTRβ1 with T_3 _failed to improve the ability of the dimer hRXRα-hTRβ1 to bind to DNA.

We used uremic plasma from four different patients to determine whether the observed inhibition of RXRα-TRβ1 binding to DNA (DR-4) was patient specific. Uremic plasma samples from all four patients inhibited RXRα-TRβ1 binding to DR-4 to various degrees (not shown). However, we could not detect any correlation between TR binding impairment and abnormalities in plasma levels of urea, creatinine or thyroid hormone.

**Figure 2:**
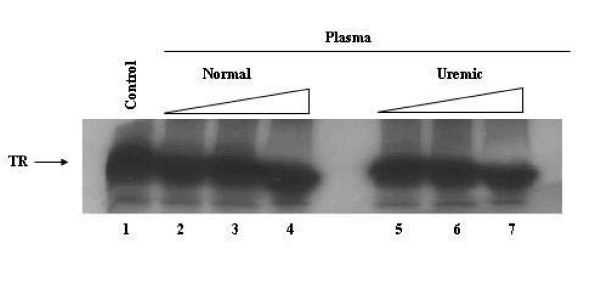
**Lack of proteolytic activity of uremic plasma on TRβ1**. Recombinant [^35^S]TRβ1 was produced by translation in reticulocyte lysate and then incubated for 30 min at 4°C without (Control – lane 1) or with increasing volumes (0.5, 1.0, 2.0 μL) of normal (lanes 2–4) or uremic (lanes 5–7) plasma. Products were analyzed by SDS-PAGE and autoradiography.

To exclude the possibility that the inhibition of hRXRα-hTRβ1 binding to DNA induced by uremic plasma could be due to proteolytic degradation of TRβ1, we incubated ^35^S-labeled TRβ1, at the same conditions as in the gel-shift experiments, with normal or uremic plasma at 4°C, for thirty minutes. ^35^S-labeled TRβ1 samples were then analyzed by SDS-PAGE. As expected, the major translation product of TRβ1 was 53 kD and incubation of theses products with normal or uremic plasma did not modify the translated [^35^S]TRβ1 (Figure 2). These results indicated an absence of uremic proteolytic activity that might be involved in the decrease of TR-RXR complex formation on DNA.

### Hemodialysis improves hTRβ1-hRXRα binding to DR-4

**Figure 3:**
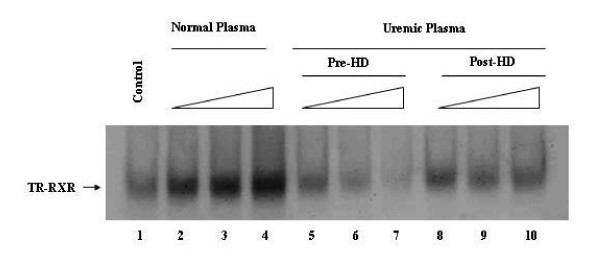
**Hemodialysis reduces the inhibitory effect of uremic plasma on hTRβ1-hRXRα binding to DR-4. **[^35^S] hTRβ1 was pre-incubated without (Control – lane 1) or with increasing volumes (0.5, 1.0, 2.0 μL) of normal (lane 2–4) or uremic plasma (patient 1) collected before (pre-HD – lanes 5–7) or after hemodialysis (pos-HD – lanes 8–10) for 30 min at 4°C. Cold DR-4 type TRE, cold hRXRα, and nonspecific DNA poly (dIdC) were subsequently added and incubated for 20 min.

It is yet unknown why uremic plasma diminished TRβ1-RXRα binding to DNA, when compared to non-uremic plasma. The observed effect could be ascribed either to a lack of some factor(s) typically present in normal plasma or to the presence of some inhibitory products present in uremic plasma. To test the second hypothesis, we analyzed the influence of uremic plasma, collected before and after hemodialysis, on TRβ1-RXRα-DR-4 complex formation using gel-shift assays. In these experiments, [^35^S] TRβ1 was incubated with normal or uremic plasma, collected before (pre-HD) or 4 h after hemodialysis (post-HD). As shown in Figure 3, uremic plasma collected before HD (lanes 5–7) decreased the binding of hTRβ1-hRXRα to DNA (DR-4), relative to normal plasma (lanes 2–4). However, when these receptors were pre-incubated with uremic plasma collected from the same patient after hemodialysis (lane 8–10), an important improvement of hTRβ1-hRXRα binding to DR-4 was observed. Although hemodialysis improved complex formation, it did not completely recover the inhibition caused by uremic plasma. Densitometry analysis demonstrated that hemodialysis increased by 50% the hTRβ1-hRXRα heterodimer binding to DNA when compared to pre-HD uremic plasma (not shown).

### Hemodialysis reduces the inhibitory effect of uremic plasma upon hVDR-RXRα binding to DNA

In view that HD improved the binding of hTRβ1-hRXRα to DR-4 response element, and that previous studies showed that uremic solutions inhibited hVDR-RXRα binding to DR-3-response element (VDRE) [26,27,29] we decided to determine whether uremic plasma collected before and after hemodialysis has the same effect on hVDR-RXRα binding to DR-3.

**Figure 4:**
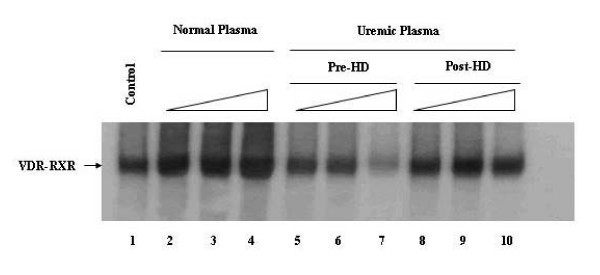
**Uremic plasma inhibits VDR-RXRα-DR-3 complex formation and hemodialysis reduces this effect. **Gel Shift experiments were performed using in vitro translated [^35^S] hVDR and cold hRXRα. [^35^S] hVDR was incubated with VD_3 _vitamin for 30 min at 4°C and then without (Control – lane 1) or with increasing volumes (0.5, 1.0, 2.0 μL) of normal (lanes 2–4) or uremic plasma, collected before (pre-HD – lanes 5–7) and after hemodialysis (pos-HD – lanes 8–10) from patient 1, for 30 min. Cold DR-3 type VDRE (5'- AGCT TC **AGGTCA **AGG **AGGTCA **GAG - 3'), cold hRXRα, and nonspecific DNA poly (dIdC) were subsequently added and incubated for 20 min.

Accordingly, a similar experiment was carried out with hVDR treated with 1,25 (OH)_2 _D_3 _vitamin (VD_3_). [^35^S]hVDR was incubated with non-uremic plasma, uremic plasma collected before and after hemodialysis. As shown in Figure 4, in comparison to control (lane1), the incubation of VDR with plasma from normal individuals increased [^35^S]hVDR-RXRα binding to DR-3 (lanes 2–4). In contrast, when [^35^S]VDR was incubated with uremic plasma collected before HD, we observed a significant reduction of hVDR-hRXRα binding to DR-3 (lane 5–7). On the other hand, when compared to pre-HD uremic plasma pre-incubation with uremic plasma collected after hemodialysis (lanes 8–10) improved hVDR-hRXRα binding to DR-3. Taken together, these results suggest that dialyzable toxins were responsible for the reduced binding of hTRβ1-hRXRα and VDR-RXRα to DNA.

### Uremic plasma does not inhibit the binding of the hPPARγ-hRXR heterodimer to DR-1

**Figure 5:**
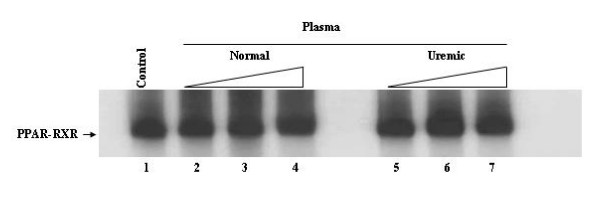
**Uremic plasma does not decrease PPARγ-RXRα-DR-1 complex formation. **Gel Shift experiments were performed using in vitro translated [^35^S] PPARγ and cold hRXRα. [^35^S] PPARγ was incubated without (Control – lane 1) or with increasing volumes (0.5, 1.0, 2.0 μL) of normal (lanes 2–4) or uremic (lanes 5–7) plasma for 30 minutes. Cold DR-1 type TRE (5'- AGCT TC **AGGTCA **G **AGGTCA **GAG - 3'), cold hRXRα, and nonspecific DNA poly (dIdC) were subsequently added and the reaction was incubated for 20 min.

Next we decided to evaluate whether uremic plasma impairs DNA binding of other members of the nuclear receptor superfamily. We performed gel shift assays using the nuclear fatty acid receptor PPARγ and its response element, DR-1. Members of the PPAR family have been shown to play an important role in obesity and the plurimetabolic syndrome [30] and insulin resistance has also been described in uremic patients [31]. In contrast to what was observed with TRβ1 and VDR, Figure 5 shows that the incubation of [S^35^] hPPARγ with uremic plasma failed to reduce the binding of PPARγ-hRXRα to DR-1 (lanes 2–4 compared to lanes 5–7). Such finding suggests that the inhibitory effect of uremic plasma does not extend to all members of nuclear receptor superfamily.

### The inhibitory activity of uremic plasma is not thermo-labile

**Figure 6:**
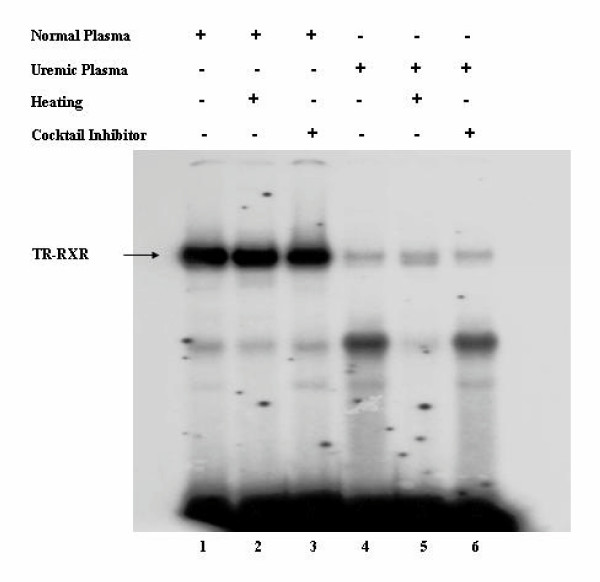
**Heating the uremic plasma does not diminish the inhibition of hTRβ1-hRXRα binding to DR-4. **Gel Shift experiments were performed using *in vitro *translated cold hTRβ1, cold hRXRα and [^32^P]DR-4. Cold hTRβ1 was pre-incubated with normal or uremic plasma, heated or not, and in the presence or absence of phosphatase inhibitor for 30 min at 4°C. [^32^P] DR-4 type TRE, cold hRXRα, and nonspecific DNA poly (dIdC) were subsequently added and the reaction was incubated for 20 min.

Our findings demonstrated that hemodialysis partially corrected the inhibition of hTRβ1-hRXRα binding to DR-4 caused by uremic plasma. We therefore hypothesized that dialyzable toxins may cause the inhibitory effect of uremic plasma on protein-DNA complex formation. To gather more information on the nature of these toxins we heated normal and uremic plasma for 5 minutes at 100°C to test if the inhibitory factor was thermo-labile. We subsequently performed gel shift assays. To control for our experimental setup we used in these experiments [^32^P] labeled DR-4 with unlabelled TRβ1-RXRα (Figure 6). Furthermore, in order to avoid any effect from plasmatic phosphatases on [^32^P] DNA, we also incubated the normal and uremic plasmas with a phosphatase inhibitor cocktail (lane 6).

Heating the plasma from normal individual did not affect the binding of the hTRβ1-hRXRα heterodimer to [^32^P] DR-4 (lane 1 compared to lane 2). As shown in prior experiments, uremic plasma significantly diminished the hTRβ1-hRXRα – [^32^P] DR-4 complex formation (lane 4). However, heating the uremic plasma did improve the binding of the hTRβ1-hRXRα heterodimer to [^32^P] DR-4 (lane 5). In addition, the effects of uremic plasma persisted in the presence of phosphatase inhibitors (lane 6).

### Uremic Ultrafiltrate Impairs T_3 _Transcriptional Activation

**Figure 7:**
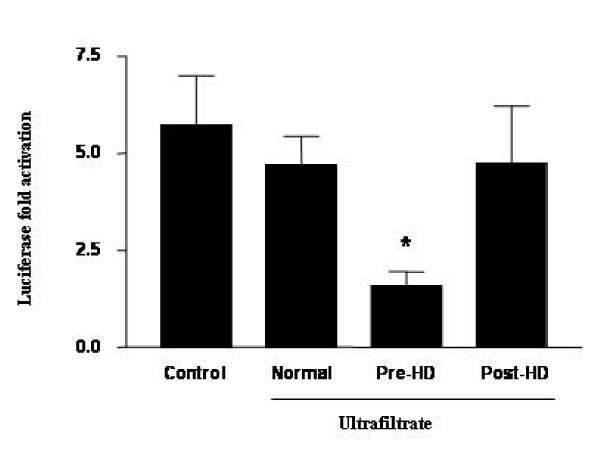
**Uremic toxin (s) impair (s) T_3_-dependent transcriptional activation in U937 cells**. A reporter construct consisting of two copies of a direct repeat thyroid response element (DR-4) 5'AGGTCAcaggAGGTCA 3' cloned upstream from the minimum thymidine kinase (TK) promoter, linked to the luciferase gene was examined in U937 cells. After electroporation, cells were transferred to fresh RPMI-1640 medium without (Control) or with normal or uremic ultrafiltrate solution, collected before (Pre-HD) or after hemodialysis (Post-HD). The cells were then plated in 12-well dish and treated with T_3 _10^-7^M. After 24 h, cells were assayed for luciferase and β-galactosidase activities. * p < 0.05 versus control and normal ultrafiltrate.

Lastly we examined if the *in vitro *inhibitory effect of uremic plasma on TR binding to DNA could affect the functions of TR as a transcription regulator in a cell based assay (Figure 7). We transfected U937 cells with cDNA encoding the human TRβ1 and a reporter gene with a DR-4 response element upstream of the firefly luciferase coding sequence. We used RPMI-1640 medium treated with ultrafiltrate (UF) solution 10-fold concentrated from normal or uremic patients collected before or after hemodialysis. As shown in Figure 7, in the absence of any UF (Control), T_3 _activated transcription by 5.7 ± 1.2 fold. When the cells were treated with UF from normal individuals, we did not observe significant changes in the transcription activation of the luciferase reporter (4.7 ± 0.74 fold; ns). Interestingly, when compared to the UF from normal individuals, UF collected Pre-HD from uremic patients reduced T_3_-dependent transcriptional activation by 66% (1.6 ± 0.35, p < 0.05). On the other hand, U937 cells treated with UF collected Post-HD had no significant effect on T_3_-dependent transcription activation (4.8 ± 1.47 fold; ns). These results suggest that uremic toxins impair T_3 _induced transcriptional activation and that hemodialysis reduces their inhibitory effect.

## Discussion

Uremia is a systemic chemical toxemia with repercussions on different organs and systems. Chronic renal failure patients demonstrate several endocrine dysfunctions, such as disturbance of thyroid hormone metabolism and are different from patients with the euthyroid sick syndrome. In the later, the conversion of T_4 _to T_3 _is reduced, but the generation of reverse T_3 _(rT_3_) from T_4 _is increased. In uremic patients, rT_3 _is typically normal [32-34]. In addition, Lim *et al*. showed thyroid hormone resistance in hemodialysis patients with significantly reduced peripheral tissue sensitivity to thyroid hormone [17]. Recent data from our laboratory indicate that in order to maintain the euthyroid state showed that uremia increases T_3 _influx across erythrocyte's membrane [35]. Taken together, these findings suggest that CRF affects thyroid function in multiple ways. The molecular mechanisms involved and the role of thyroid hormone receptor in this dysfunction, however, are not fully understood.

In the present study we observed that uremic plasma impaired the ability of TRβ1 and VDR heterodimers (TRβ1-RXRα and VDR-RXRα) to bind to DNA (DR-4 and DR-3 respectively), whereas that the ability of PPARγ-RXRα to bind to DNA (DR-1) was not altered. Interestingly, there was no correlation between the inhibitory activity and the plasma levels of urea, creatinine, parathyroid and thyroid hormone of the patients enrolled in this study. However, the small number of patients precludes any definitive conclusions.

To investigate whether these findings were secondary to the presence of uremic dialyzable toxins, we compared the effect of uremic plasma collected before and after hemodialysis, on TRβ1-RXRα or VDR-RXRα binding to DNA. Our results showed that the inhibitory effect of uremic plasma was significantly reduced by hemodialysis, suggesting that dialyzable toxins were in fact involved. We did not identify which toxin is responsible for this effect, but our results suggest the presence of thermo-resistant molecule(s). Further analyses of these dialyzable toxins are currently being conducted to identify and characterize the molecules responsible for this inhibitory effect.

The mechanisms responsible for our findings are not clear. In uremic syndrome the reduced clearance of many toxins plays a key role in this pathogenesis. Although VDR degradation has been suggested in renal failure [36], the uremic inhibition of TRβ1-RXRα binding to DNA could not be explained by proteolytic activity of uremic plasma since our SDS-PAGE did not show any uremic plasma-dependent degradation of TRβ1.

Our results are in agreement with other studies, which have shown that uremic toxins are involved in VD_3 _resistance observed in patients with chronic renal failure [37]. Uremic ultrafiltrates derived from hemo or peritoneal dialyzed patients have been shown to inhibit the interaction of VDR with DNA [27,28]. Further studies showed that the VDR complex formation on different types of VDREs can be reduced by uremic solutions collected from patients after hemo or peritoneal dialysis [28]. Our results allow us to speculate on the possibility of a common inhibitory mechanism involving the same uremic toxin(s) inhibiting both TR-RXR-DR-4 and VDR-RXR-DR-3 complex formation.

To evaluate whether uremic toxins also affect other members of the nuclear receptor family, we studied the effect of uremic plasma on PPARγ-RXRα binding to DR-1. Contrary to what we observed with TRβ1 and VDR, pre-incubation of PPARγ with uremic plasma did not influence PPARγ-RXRα binding to DNA. This result suggests that the inhibition of protein-DNA complex caused by uremic plasma occurs only with some nuclear receptors. Taken together, our results indicate that uremic toxins exert their inhibitory effect by acting specifically on TRβ1 and VDR heterodimers.

The molecular mechanism involved in this phenomenon is not clear. The fact that PPARγ-RXRα heterodimer was not affected by uremic plasma suggests that these toxins do not interact directly with RXRα. Another possible model to explain the effects of the toxins from uremic plasma on the binding of TR to DNA would be a direct action on DNA that would block its interaction with TRβ1 and VDR heterodimers. However, even though we used the DR-1 in PPARγ assay, in contrast to DR-4 (TRE) and DR-3 (VDRE), this hypothesis is not strongly supported by the results from this study, as PPARγ-RXRα heterodimers bind normally to DNA in the presence of uremic plasma. Another alternative that can not be excluded is an inhibitory effect of the uremic toxin on the surface of TR and VDR DNA binding domain (DBD), disrupting its ability to binding to DNA. Patel *et al. *attributed to the formation of Schiff bases between "reactive aldehydes" and lysine residues of the DBD of the VDR to explain the inhibitory effect of the uremic ultrafiltrate on the binding of VDR to DNA [26]. Nevertheless, in another study, point mutagenesis of different lysine residues in the DBD could not confirm this idea [28]. In addition, we should consider that the uremic toxins can interact with TR and VDR, causing structural conformational changes on these receptors, consequently, impairing heterodimers formation.

We attempted to demonstrate the physiological relevance of these results by examining the effect of uremic toxins on T_3 _transcriptional activation. Our results showed that uremic ultrafiltrate collected before hemodialysis inhibited T_3_-induced transcriptional activation, confirming the *in vitro *findings. Conversely, in the presence of ultrafiltrate collected after hemodialysis, the transcriptional activation induced by T_3 _was similar to the control group treated with ultrafiltrate collected from normal individuals. Therefore, we hypothesize that dialyzable toxins are responsible for the resistance to T_3 _action documented in CRF patients.

In summary, uremic toxins circulating in the plasma of CRF patients selectively reduced the binding of TRβ1-RXRα to DNA and impaired the TRβ1 transcriptional activation mediated by T_3_. Moreover, hemodialysis partially corrected this inhibitory effect, suggesting the presence of a dialyzable toxin. Since TRβ1 functions as a heterodimer with RXRα, these findings might explain some features of hypothyroidism and thyroid hormone resistance commonly found in CRF patients. Future studies are necessary to identify the toxins and further characterize the mechanisms involved in resistance to T_3 _action in CRF patients.

## Materials and methods

### Patients and Clinical Procedures

Four patients from the chronic dialysis program of Soclimed Dialysis Clinic were enrolled in our study. All patients were men whose age ranged from 19 to 43 years with the mean age being 34 years. They appeared well nourished and clinically and laboratorial euthyroid; none had a history of thyroid disease, thyroid hormone therapy, treatment with amiodarone or clinically detectable goiter. Etiology of their chronic renal failure was as follows: chronic glomerulonephritis (2); hypertension (1); reflux nephropathy (1). Mean plasma urea level was 178 ± 44.8 mg/dL (120 to 233 mg/dL), while that of creatinine was 12.6 ± 2.7 mg/dL (9.9 to 16.3 mg/dL). Patients were on hemodialysis 3 times a week, during 4 hours using a 1.8 m^2 ^Fresenius^® ^Polysulfone filter. Normal control subjects consisted of three healthy men, age ranging from 23 to 41 years, with the mean age of 32 years. The experimental protocol was approved by the Human Rights in Research Committee of the University of Brasilia and all patients and normal individuals gave their informed consent.

For the *in vitro *DNA binding assay, uremic plasma was collected immediately before and after 4 h of hemodialysis, aliquoted into 20 μL samples and stocked at -20°C. Uremic ultrafiltrate (UF) was also collected pre and post 4 h hemodialysis. Lyophilisation was used to concentrate ultrafiltrate samples. The lyophilisates were re-suspended in bidistillated water to a 10-fold concentrated solution, as effects of the UF were not detectable at lower concentrations (1 fold, 2.5 fold, 5 fold concentrated; not shown). Samples were subsequently desalted by filtration using Centricon 3 filters. Following centrifugation, the pellet was re-suspended in RPMI-1640 medium, (10% newborn bovine serum; 2 mM glutamine; 50 units/mL penicillin; 50 μg/mL streptomycin) and pH corrected to 7. Normal UF was collected from control plasma of normal individuals. The treatment solution was prepared in the same manner as the uremic solution. All experiments were performed with the uremic sample from the same patient that showed the strongest inhibitory effect.

### Gel shift binding assay

Gel shift assays were used to evaluate the binding of ^35^S-labeled TR synthesized in reticulocyte lysate on 600 fmoles of unlabeled DR-4 (5'-AGCT TC AGGTCA CAGG AGGTCA GAG -3') and inverted palindrome – F2 (5'-TTC TGACCC CATTGG AGGTCA GAG -3'); ^35^S-labeled VDRs to unlabeled DR-3 5'- AGCT TC AGGTCA AGG AGGTCA GAG - 3') and ^35^S-labeled PPARγ to unlabeled DR-1 (5'- AGCT TC AGGTCA G AGGTCA GAG - 3'). Sensitivity and specificity of this assay have been previously characterized [23]. Briefly, the labeled protein will migrate in the nondenaturating polyacrylamide gel only when bound to DNA. Additionally, gel shift were also performed using unlabeled synthesized TRs and ^32^P-labeled DR-4.

*In vitro *receptor synthesis was performed using plasmids encoding hTRβ1 hRXRα, and hPPARγ [38] and hVDR [39] with the TNT-coupled Reticulocyte Lysate System (Promega, Madison, WI) containing a methionine-free aminoacid mixture, and either 20 μM cold methionine or ^35^S-labeled methionine. DNA plasmid (0.2–2 μg) was added to TNT Quick Master Mix and incubated in 50 μL for 90 min at 30°C. To confirm efficiency of the translation reaction ^35^S-labeled translated proteins were analyzed by sodium dodecyl sulfate gel electrophoresis (SDS-PAGE). The TR-DNA complex was visualized by labeling reticulocyte lysate-translated receptors with ^35^S or by using labeled DNA with ^32^P using polynucleotide kinase. Prior to incubation with DNA, the reticulocyte lysate-translated receptors were treated with TRβ1, VDR and PPARγ receptors ligands (T_3_, 1,25-dihydroxy-vitamin D_3_; and 9-*cis*-retinoic acid respectively) for 30 min at 4°C. Labeled nuclear receptor plus ligands were then incubated in different volumes (0.5, 1 and 2 μL) of uremic or normal plasma for 30 min at 4°C. Following plasma exposure, the nuclear receptors were incubated for another 20 min at room temperature (20–30°C) in a solution containing 2 μg non-specific DNA poly (dIdC) (Pharmacia LKB, Piscataway, NJ), cold specific response element (10 ng/reaction), nonradioactive RXRα and a binding buffer in a 20 μL reaction as previously described [23]. When using non-labeled nuclear receptors the gel shift experiment was performed using ^32^P-DR-4 (2000–5000 cpm). A phosphatase inhibitor cocktail 2 (Sigma, P 5726) was added when the DNA was labeled (^32^P-DR-4). The binding buffer contained 0.2 mM Na_2_HPO_4_, 0.2 mM NaH_2_PO_4_, 1 mM MgCl_2_, 0.5 mM EDTA, and 5% glycerol. Final samples were loaded on 5% nondenaturing polyacrylamide gel, previously run for 30 min at 200 V. To separate the protein-DNA complexes, the gel was run at 4°C for 90–180 min at 240 V, using a running buffer (pH 7.5 for 10X stock at room temperature) containing 6.7 mM Tris-base, 1 mM EDTA, and 3.3 mM Sodium Acetate. The polyacrylamide gel was dried at the end of electrophoresis and autoradiographed.

### Cell Culture, Transfections and Reporter Gene Assays

Human promonocyte U937 cells were maintained in culture as previously described [38]. For transfection assays, cells were collected by centrifugation and resuspended in transfection solution (0.5 mL/ 1.5 × 10^7 ^cells) containing PBS, 100 mM calcium and 0.1% dextrose and mixed with 2 μg of human TRβ1 expression vector, 4 μg of the luciferase (Luc) reporter and 500 ng control β-galactosidase vector. The reporter plasmid contained a synthetic TR response element containing two copies of DR-4 cloned immediately upstream of a minimal thymidine kinase (tk) promoter (-32/+45) linked to luciferase coding sequences [38]. The cells were transferred to a cuvette and electroporated using a Bio-Rad gene pulser at 300 V and 960 μF.

Immediately after electroporation, the cells were transferred to fresh RPMI-1640 medium treated without or with normal or uremic ultrafiltrate solution (10 fold concentrated) collected before or after hemodialysis. Cells were then plated in 12-well dish and treated in triplicates with T_3 _10^-7^M or ethanol (vehicle). After 24 h, cells were collected by centrifugation, lysed by the addition of 150 μL 1X lysis buffer (Promega) and assayed for luciferase (kit from Promega Corp.) and β-galactosidase (kit from Tropix, Inc., Bedford, MA) activities. All transfection experiments were performed at least three times.

### Statistics

Data were analyzed by Kruskal-Wallis test followed by Dunn's Multiple Comparison Test when applicable. P < 0.05 was considered statistically significant.

## Competing interests

The author(s) declare that they have no competing interests.

## Authors' contributions

G.M.S. carried out all the experiments and prepared the manuscript. C.J.A.B.P. helped with cells culture and manuscript preparation. A.C.S. and M.C.S. selected and monitored the patients and collected all uremic plasma samples enrolled in this study. L.A.S. and R.C.J.R. participated in design the experiment. N.L. helped with analysis of the data and in drafting the manuscript. F.A.R.N. conceived the study and participated in its design and coordination. All authors read and approved the final manuscript.
